# Tau strains shape disease

**DOI:** 10.1007/s00401-021-02301-7

**Published:** 2021-04-08

**Authors:** Jaime Vaquer-Alicea, Marc I. Diamond, Lukasz A. Joachimiak

**Affiliations:** 1 Neuroscience Graduate Program, University of Texas Southwestern Medical Center, Dallas, TX 75390 USA; 2 Center for Alzheimer’s and Neurodegenerative Diseases, Peter O’Donnell Jr. Brain Institute, University of Texas Southwestern Medical Center, Dallas, TX 75390 USA; 3 Department of Biochemistry, University of Texas Southwestern Medical Center, Dallas, TX 75390 USA

**Keywords:** Tau, Propagation, Folding, Tauopathy, Strains, Amyloid, Prion, Aggregation, Diagnosis, Therapeutics, Self-assembly, Polymorph

## Abstract

Tauopathies consist of over 25 different neurodegenerative diseases that include argyrophilic grain disease (AGD), progressive supranuclear palsy (PSP), corticobasal degeneration (CBD), and Pick’s disease (PiD). Tauopathies are defined by brain accumulation of microtubule-associated protein tau in fibrillar aggregates, whose prevalence strongly correlates with dementia. Dominant mutations in tau cause neurodegenerative diseases, and most increase its aggregation propensity. Pathogenesis of tauopathies may involve pathological tau conformers that serve as templates to recruit native protein into growing assemblies and also move between brain cells to cause disease progression, similar to prions. Prions adopt pathological conformations, termed “strains,” that stably propagate in living systems, and create unique patterns of neuropathology. Data from multiple laboratories now suggest that tau acts as a prion. It propagates unique strains indefinitely in cultured cells, and when these are inoculated into mouse models, they create defined neuropathological patterns, which establish a direct link between conformation and disease. In humans, distinct fibril structures are associated with different diseases, but causality has not been established as in mice. Cryo-EM structures of tau fibrils isolated from tauopathy brains reveal distinct fibril cores across disease. Interestingly, the conformation of the tau monomer unit within different fibril subtypes from the same patient appears relatively preserved. This is consistent with data that the tau monomer samples an ensemble of conformations that act as distinct pathologic templates in the formation of restricted numbers of strains. The propensity of a tau monomer to adopt distinct conformations appears to be linked to defined local motifs that expose different patterns of amyloidogenic amino acid sequences. The prion hypothesis, which predicts that protein structure dictates resultant disease, has proved particularly useful to understand the diversity of human tauopathies. The challenge now is to develop methods to rapidly classify patients according to the structure of the underlying pathological protein assemblies to achieve more accurate diagnosis and effective therapy.

## Introduction

Tauopathies are a large group of neurodegenerative diseases, unified by accumulation in the brain of fibrillar aggregates of the protein microtubule-associated protein tau (MAPT). While these diseases are all linked to the deposition of tau, the morphology of the tau aggregates varies by disease [51]. Furthermore, there is poor correlation between clinical phenotypes and neuropathology [73]. New work on tau suggests that it behaves as a prion: it converts from a soluble, monomeric state to one that self-propagates aggregates rich in beta-sheet structure [105]. These tau assemblies stably maintain unique conformations in vivo that induce or “seed” the native monomer to oligomerize into amyloid aggregates [67, 104]. Recent structural studies of tau fibrils isolated from patient samples have revealed conformations unique to each of several tauopathies [106]. Human and animal studies strongly suggest that tau strains spread distinct tauopathies through the brain[43]. The concept of prion strains usefully frames the question of how tau’s distinct conformational states might cause distinct diseases. This review will discuss why knowledge of tau strains could bridge the current gap between clinical presentation and neuropathology, in which an assigned antemortem diagnosis often is not confirmed by subsequent neuropathological analysis. Importantly, insight into the initial formation and subsequent propagation of distinct strains could inform future diagnostic and therapeutic strategies.

## Tau

**Fig. 1:**
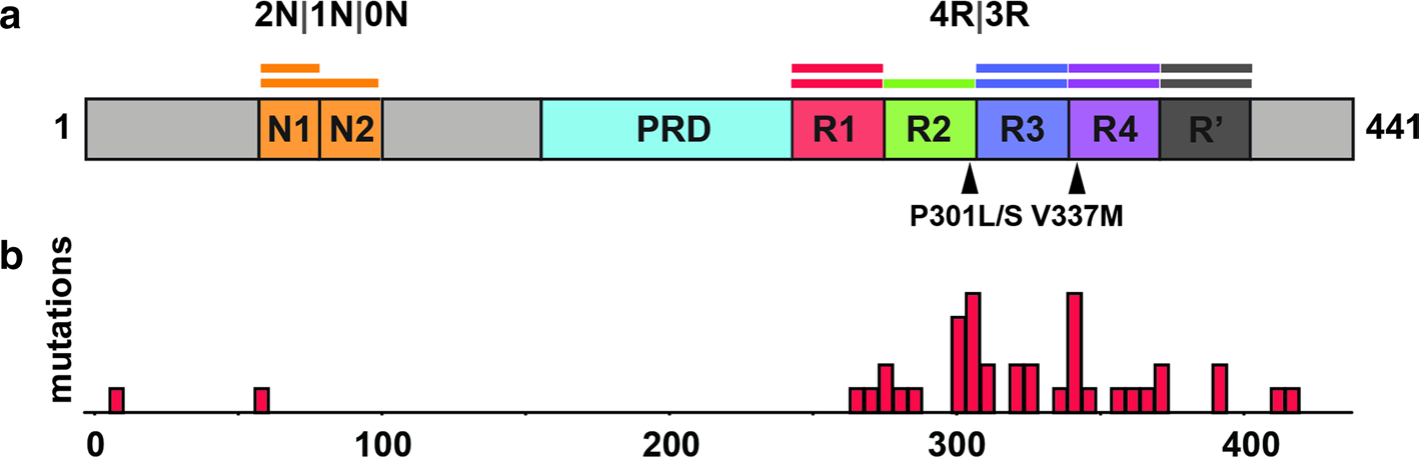
Tau protein. **a** Domain organization of tau brain isoforms. Schematic of the 441 residue 2N4R tau isoform highlighting the domains (N1, N2 and R2) which define the isoforms. The tau repeats are colored red (R1; residues 244–274), green (R2; residues 275–305), blue (R3; residues 306–336), purple (R4; residues 337–368) and dark grey (R’; residues 369–400). The proline-rich domain (PRD) is colored in light blue and the N1 and N2 domains are colored in orange. Two key disease-associated mutations are highlighted by arrows: Proline301 to serine or leucine mutations and valine337 to methionine. **b** Disease-associated mutation frequencies found in human tauopathies. Most mutations are found within the repeat domain

The human gene encoding the microtubule-associated protein tau (*MAPT*), is located on chromosome 17q31 [90]. It encodes 16 exons, of which exons 2, 3 and 10 are alternatively spliced [6, 90]. In the human central nervous system, it may exist as six isoforms (0N3R, 0N4R, 1N3R, 1N4R, 2N3R, and 2N4R, detailed in Fig. 1a) and is highly abundant in neurons [45, 50, 83], whereas in the periphery a longer form is expressed [25, 48]. In the developing brain, isoforms mostly lack exon 10, which encodes the second of four highly conserved microtubule-binding repeats (Fig. 1a; R1, R2, R3 and R4) followed by a fifth less conserved repeat (Fig. 1a; R’) [49]. The adult human brain expresses both 4R and 3R isoforms at near equivalent proportions [45, 55]. The consequence of these expression patterns is not understood.

A causal role of tau was established in neurodegeneration when dominant mutations in *MAPT* were determined to cause familial Frontotemporal dementia with Parkinsonism linked to chromosome 17 (FTDP-17) [56, 96, 116]. The majority localized to the repeat domain (Fig. 1b). Most mutations decrease microtubule binding and increase aggregation propensity, both in vivo and in vitro, and transgenic mouse models with these forms of mutant tau exhibit neurodegenerative phenotypes in association with tau fibril formation [4, 46, 79]. Mutations that do not increase aggregation may increase protein levels or alter isoform ratios, favoring inclusion of exon 10, and promoting four-repeat tau [115, 119].

## Diversity of tauopathies and gaps in our knowledge

**Fig. 2:**
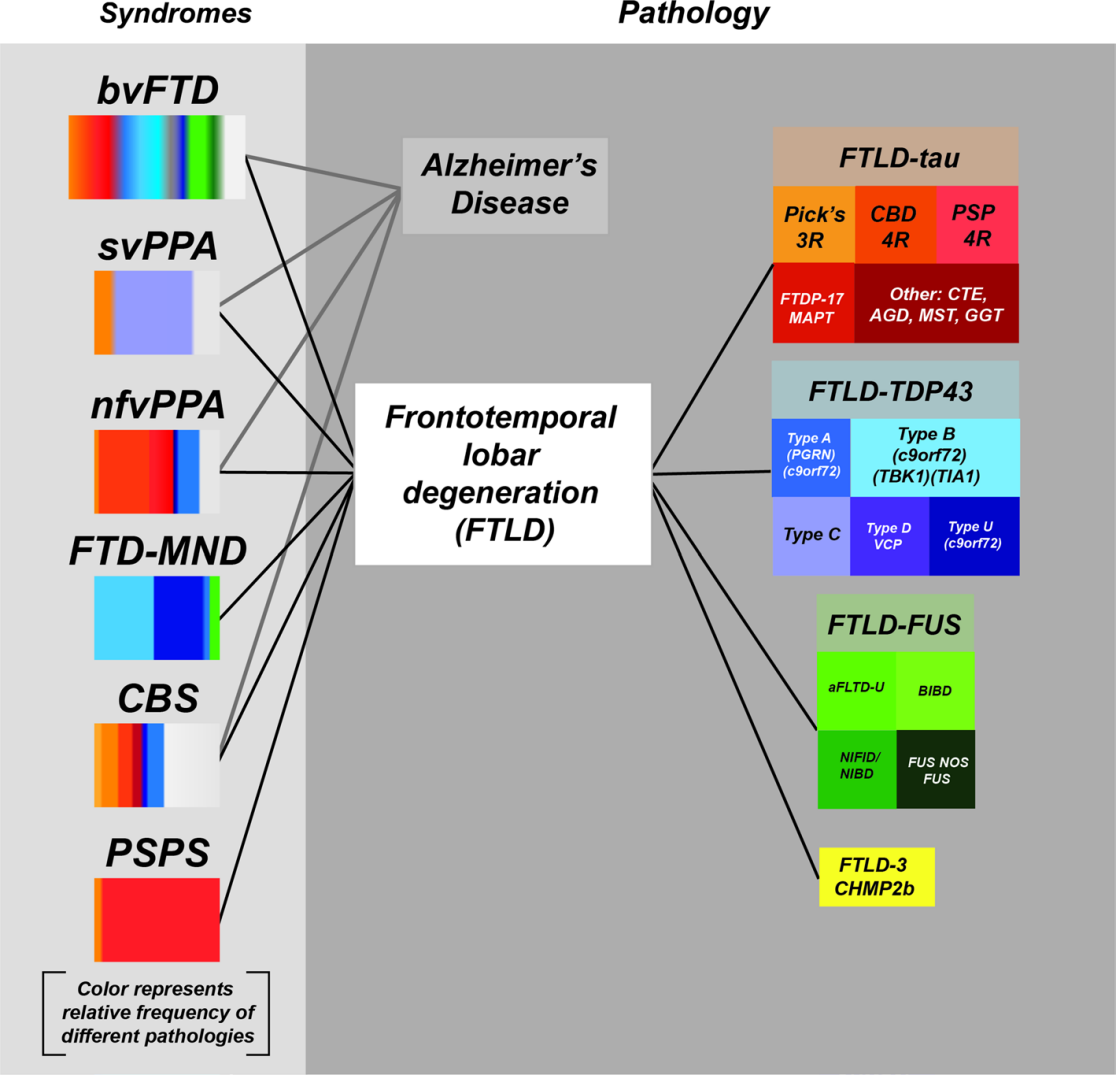
Relationship between clinical syndromes and neuropathology. Illustration of the association of different clinical syndromes with the deposition of specific inclusions of proteins including tau as determined by neuropathology. Each disease, defined by neuropathology of specific proteins, is colored differently. The fractional percentage of protein deposition in each clinical syndrome is estimated from the literature. (Figure is adapted from a slide shared by Dr. William Seeley, UCSF)

Neurodegenerative tauopathies are defined by deposition of abnormal tau as ordered beta-sheet-rich fibrils. Individuals with tauopathy often display symptoms consistent with Alzheimer’s syndrome (AS), frontotemporal dementia (FTD), corticobasal syndrome (CBS) or progressive supranuclear palsy syndrome (PSPS). However, within these clinical presentations there is considerable neuropathological variation, including the involvement of proteins other than tau [62, 89]. Hence, while clinical symptoms reflect dysfunction in specific brain regions that have succumbed to pathology, the considerable anatomical and symptom overlap among tauopathies, and the involvement of other amyloid proteins makes it difficult to reliably determine antemortem the underlying proteinopathy based on presentation alone [10, 58]. For example, a patient may present with symptoms of the clinical syndrome known as behavioral variant FTD (bvFTD), yet the underlying disease may be due to one of more than ten possible neurodegenerative pathologies grouped under the umbrella term of frontotemporal lobar degeneration (FTLD) (Fig. 2).

The gold standard for classifying neurodegenerative diseases is neuropathology. However, the lack of correspondence between clinical syndromes and neuropathological diagnosis suggests a fundamental gap in understanding of these diseases [73]. The recent discovery that different tau strains are sufficient to induce diverse neuropathological outcomes in mouse models and the atomistic description of tau fibril polymorphs (structural variants) associated with unique tauopathies suggests that defining the relationship between tau strains and clinical syndromes will let us diagnose and thus treat tauopathies more effectively. This is entirely analogous to how we can now parse cancer subtypes based on genetic and epigenetic features, which in turn guides diagnosis and therapy.

## Clinical syndromes associated with tau neuropathology

AD and FTLD pathologies may be discriminated molecularly and histologically but they often present with similar diagnostic features [73]. Age-related phenotypic variations further complicate the antemortem diagnosis of these diseases.

The first four syndromes are collectively termed FTD and encompass behavioral and language phenotypes (Fig. 2).

**bvFTD** (behavioral variant) is the most common variant of FTD. It refers to a disorder of conduct, judgement, self-control, and socialization.

**nfvPPA** (non-fluent variant) of primary progressive aphasia (PPA). The syndrome is characterized by difficulties in the production and grammatical structure of speech.

**svPPA** (semantic variant) of PPA is characterized by focal word loss during spontaneous speech.

**lvPPA** (logopenic variant) PPA is characterized by slow or hesitant speech without problems with articulation but with momentary difficulties in word finding. Impaired sentence comprehension and naming are also present.

**CBS** (corticobasal syndrome) and **PSPS** (progressive supranuclear palsy syndrome) are motor syndromes that may reflect underlying tauopathy. CBS features unilateral rigidity, apraxia, and alien hand phenomena. PSPS prominently features axial rigidity, bradykinesia, vertical gaze palsy and dysphagia.

**AS** (Alzheimer’s syndrome) is typically characterized by a progressive amnestic phenotype with executive and visuospatial dysfunction.

## Neuropathology of tauopathies

**Fig. 3:**
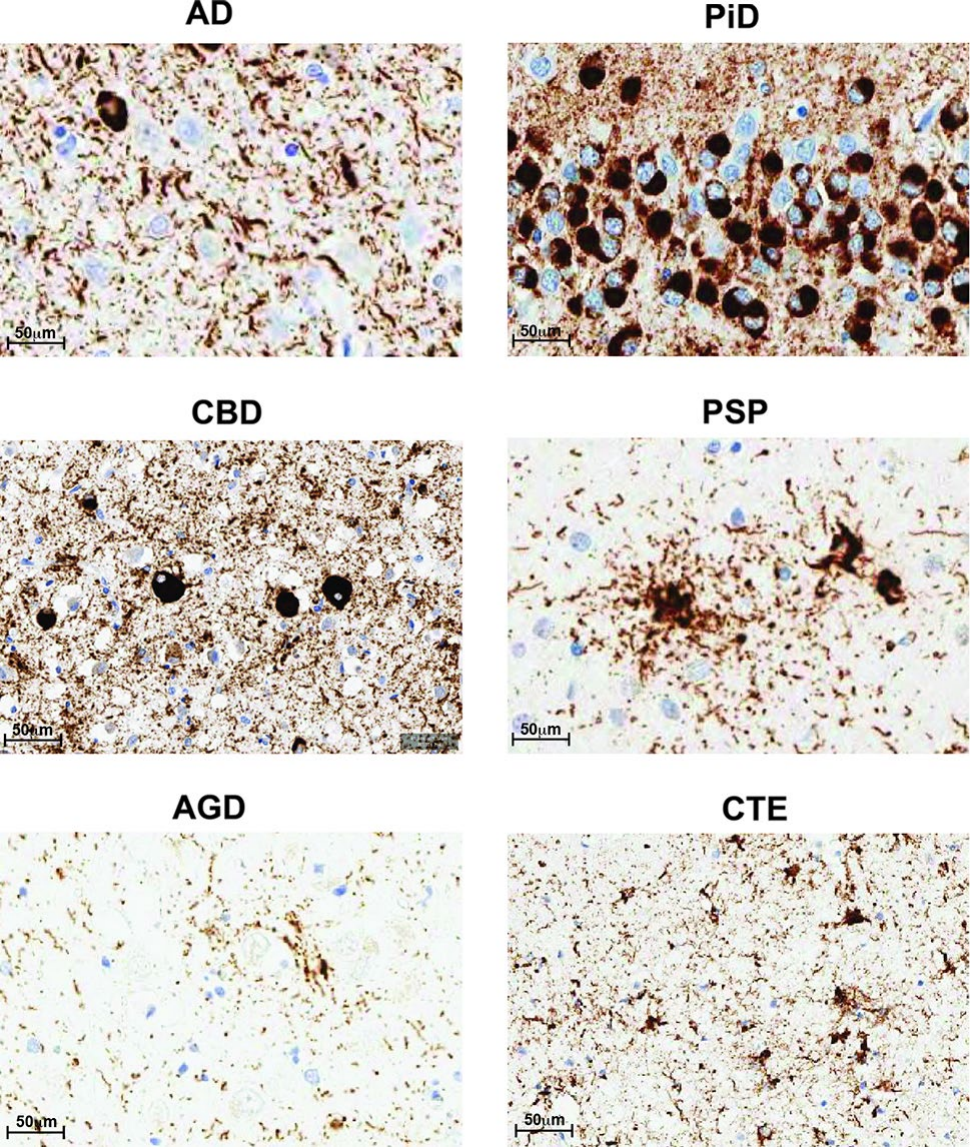
Neuropathology of tauopathies. Representative IHC staining using AT8 on brain sections from different human tauopathy patients AD, CBD, PSP, AGD, PiD and CTE

Primary tauopathies are those in which tau deposition is the most pronounced pathological finding. Neuropathological tau phenotypes are most often classified by their anatomical distribution, cell type involvement, and the protein isoforms deposited (Fig. 3, Table 1) [74, 75]. The most prominent primary tauopathies include corticobasal degeneration (CBD), progressive supranuclear palsy (PSP), Pick’s disease (PiD) and argyrophilic grain disease (AGD) [51, 58, 115]. Alzheimer’s disease (AD), the most studied tauopathy, features aggregated tau in the form of neurofibrillary tangles (NFTs), linked to prior deposition of amyloid beta protein aggregates, and is thus classified as a secondary tauopathy (Fig. 3) [51]. Secondary tauopathies may also result from environmental exposure such as trauma in the case of chronic traumatic encephalopathy (CTE) [41].

**Table 1:** Neuropathological diagnosis

Neuropathological diagnosis	Anatomical distribution	Neuropathological hallmark	Tau isoform
AD	Neocortex and limbic regions	Neurofibrillary tangles (NFTs), neuropil threads and dystrophic neurites	3R + 4R
PiD	Frontal, temporal, and parietal lobes	Cytoplasmic spherical structures, termed Pick Bodies	3R
CBD	Focal atrophy of superior frontal gyrus and parietal lobe	Small-NFTs, corticobasal bodies (Fig. 3b) and diffuse granular tau inclusions. Occasional Pick bodies are observed. The most specific pathology is the astrocytic plaque which appears as a circular or ring-shaped collection of argyrophilic tau-positive cell processes	4R
PSP	Subthalamic nucleus, superior cerebellar peduncle and hilum of cerebellar dentate nucleus	Globose NFTs, tufted astrocytes (Fig. 3c) and oligodendroglial coiled bodies	4R
AGD	Amygdala, limbic cortex, mesial temporal lobe, and temporal neocortex	Small dot-like spindle-shaped structures, termed grains. Oligodendroglial coiled bodies and pre-tangles	4R
CTE	Varying degrees of atrophy in frontal and temporal lobes. Often found in the depths of sulci	NFTs (Fig. 3f) and astrocytic tangles	3R + 4R

At the molecular level, tauopathies may be classified by the degree of incorporation of 3R and 4R isoforms into detergent-insoluble material from brains of individuals [74]. At the histological level, characteristic patterns of atrophy and tau pathology define each disease (Table 1). While AD is considered a separate entity, PSP, CBD, AGD, PiD and CTE fall into the category of FTLD pathologies related to tau (FTLD-tau) which distinguishes them from FTLDs associated with TAR DNA-binding protein (TDP-43), fused in sarcoma (FUS) or charged multivesicular body protein 2B (CHMP2B).

## Prion strains

Hundreds of papers in the last decade have investigated the idea that tau and other amyloid-forming proteins might function as prions. The notion that a neurodegenerative disease could be mediated by propagation of a unique protein conformation originated with the discovery that the key component in prion disease infectivity was prion protein (PrP). The discovery of a protein-based infectious agent led to the Nobel prize in 1997 for Stanley Prusiner. Prions represented a stunning new biological concept wherein a protein transmitted pathological information in an infectious manner by serving as a template to corrupt native protein and thereby self-replicate. The most notable prion diseases are bovine spongiform encephalopathy (BSE), scrapie of sheep and Creutzfeldt–Jakob disease (CJD) [22, 23, 126].

Prions have been extensively characterized biochemically and by experimental transmission in vivo. In the absence of high-resolution structures—which are difficult to obtain using classical structural biology methods, prions associated with different diseases are commonly detected and compared by physicochemical analyses. This includes resolving the aggregate core by limited proteolysis, or comparing the solubility of aggregates in non-ionic detergents or sedimentation properties in sucrose gradients [35, 100]. A recent cryo-electron microscopy (cryo-EM) structure of PrP-based prion fibrils isolated from hamster brains adopt a parallel in-register intermolecular beta-sheet and connecting chains similar to other amyloid fibrils isolated from tauopathy and alpha-synucleinopathy tissues [76]. Alternatively, prions are characterized by experimental transmission into animal models which may give rise to characteristic incubation times, disease phenotypes, and distribution of pathological lesions in the brain [13, 35].

When distinct forms of prion disease were characterized it became apparent that pathogenic PrP adopts different conformations, each responsible for a different disease, and stably transmissible over time between animals [15, 37, 107]. In summary, distinct pathogenic conformers, or strains of PrP explain well the variability of prion diseases.

## Other prion proteins

Prusiner originally predicted that there might be multiple proteins that form prions based on the presence of amyloid fibrils in both scrapie and AD preparations [97]. Several groups subsequently observed that other amyloid-forming proteins such as amyloid beta, alpha-synuclein, huntingtin, and tau had similar template-based aggregation characteristics in vivo [44, 117, 118, 125]. In the 1990s, AD brain inoculation of AD lysates into primates hinted at infectious material in this disorder [9]. Walker and colleagues then observed that inoculation of bAPP-transgenic mice (tg2576) with AD brain homogenate induced b-amyloid pathology [66], and followed up this work in collaboration with Jucker [63, 85, 128]. And recent neuropathological studies of patients who developed CJD following administration of cadaveric pituitary extract indicated that amyloid beta pathology might also be transmitted between humans [29, 60, 98, 101]. Observations regarding the propagation of pathology have now been extended to other proteins including alpha-synuclein [27, 72, 80, 82], huntingtin [18, 61] and tau [20, 57, 104].

Despite the many advances in biochemistry and molecular genetics of amyloid-forming proteins, the origins of phenotypic diversity and the molecular basis of progressive neurodegeneration remained mysterious. Studies of amyloid beta revealed that a monomer could form multiple distinct fibril structures in vitro [95], and that injection of pure amyloid beta fibrils into a vulnerable transgenic mouse initiated extracellular amyloid beta deposition [85]. Moreover, conformers of amyloid beta fibrils could be transmitted by seeded conversion into brains of two mouse models of amyloid beta pathology [52]. The development of ligands for amyloid pathology has now allowed for the post-mortem discrimination of conformers of amyloid beta pathology in patients with diverse lesions, and between subjects with distinct clinical phenotypes [99, 129].

In 2009, two groups evaluated tau prion activity. Tolnay and colleagues observed that inoculation of brain lysates containing pathological tau derived from a tauopathy mouse model induced the intracellular aggregation and apparent spread of tau aggregates from the site of injection [20]. Concurrently, the Diamond laboratory discovered that exposure of cultured cells expressing full-length tau to extracellular fibrils triggered aggregate uptake that in turn triggered intracellular aggregate formation and subsequent transfer between co-cultured cells [39]. It became clear that tau might have "infectious" properties, at least from the standpoint of pathology transferred from the outside to the inside of a cell. With the development of novel cell-based detection systems (termed "biosensors") the Diamond lab subsequently determined that tau-mediated seeding activity correlates with disease progression and anticipates classical pathological markers in mouse models of tauopathy and in Alzheimer’s disease [40, 53, 54, 68].

While these observations provided ideas regarding disease progression, a major question has been the origin of diversity of neuropathological phenotypes in tauopathy. The concept of a prion strain has provided a critical framework. A strain is a self-replicating conformer that creates unique, transmissible pathological outcomes. A clue that this might underlie tauopathies came in 2013 when the Goedert lab inoculated human tauopathy lysates from PSP, CBD, and AGD into brains of ALZ17 mice, which express the longest brain isoform of human tau (2N4R). Patterns were described that were reminiscent of those seen in human pathology[19]. However, it remained unclear if aggregated tau vs. other disease-specific factors created these distinct pathological phenotypes, and, further these studies did not biochemically determine the inoculated preparations as unique protein conformers.

In 2014, Sanders et al. determined that tau is a *bone fide* prion that can be formed in vitro and creates unique strains[104]. An aggregation-prone mutant (P301L/V337M) of the repeat domain of tau fused to yellow fluorescent protein (RD-YFP) was expressed in HEK cells. When exposed to recombinant tau fibrils, distinct inclusion patterns formed and could be isolated and propagated indefinitely as unique clones via mother–daughter transfer, or via inoculation of naïve cells. Clonal tau strains had distinct seeding activity, detergent solubility, and pronase digestion patterns indicative of unique structures. Tau strains extracted from a given cell line were transmissible to naive cells by inoculation, indicating they were caused by tau, and were not a feature of the original clonal cells. Tau strains induced unique neuropathology patterns in vivo after inoculation of a mouse model expressing full-length human tau containing a disease-associated mutation (P301S). The unique patterns of neuropathology were faithfully transmitted across three generations of mice. In summary, infectious strain properties of tau were maintained in a synthetic cell system based on RD-YFP, and a mouse model based on full-length human protein. Because these studies created an infectious form of tau from recombinant protein, with strain properties linked to unique, and transmissible neuropathological outcomes in vivo, the authors considered it most appropriate to refer to tau as a prion.

**Fig. 4:**
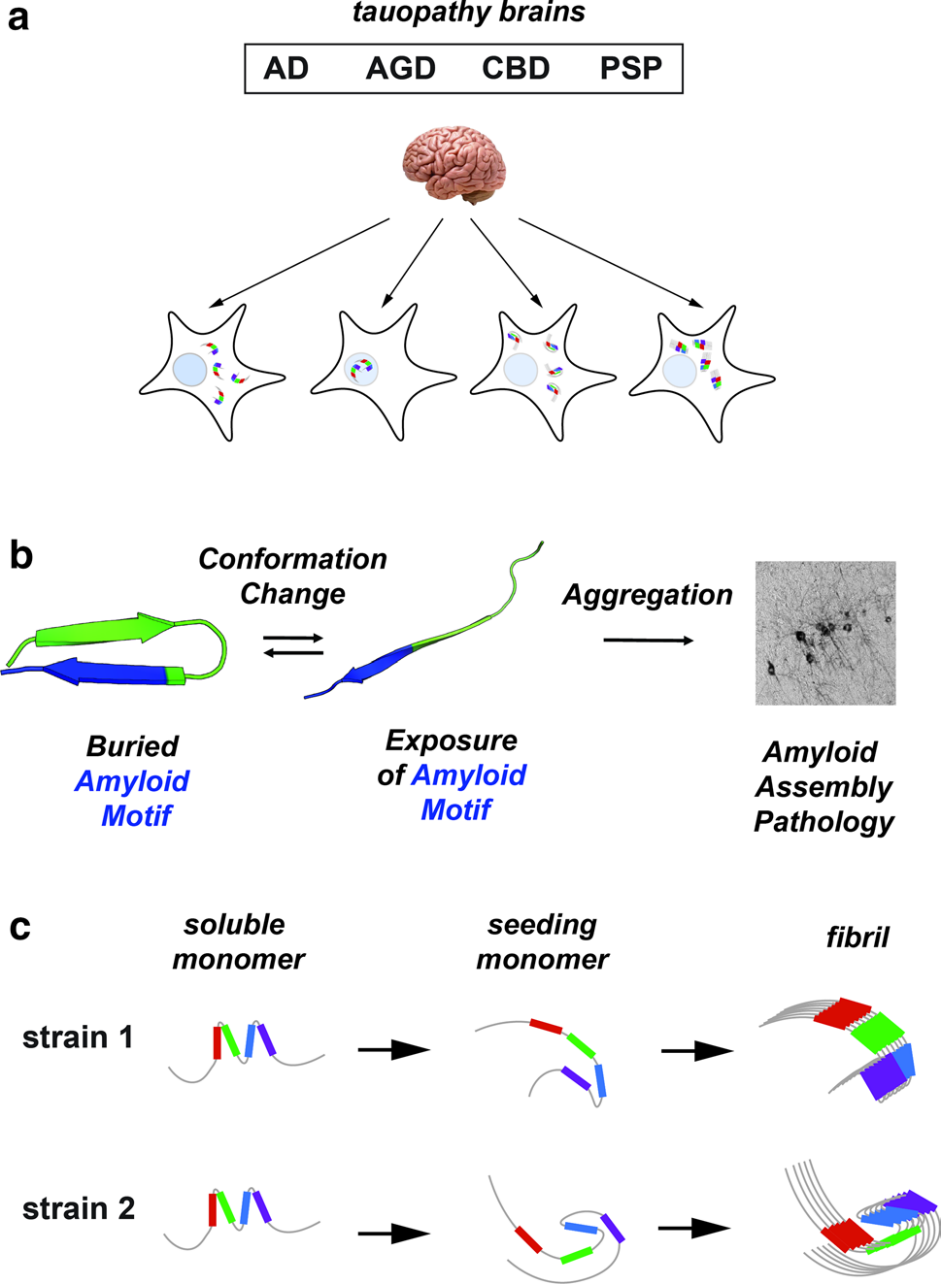
Propagation of tau strains. **a** Schematic illustrating biosensor-based detection of tau seeds derived from different tauopathies (AD, AGD, CBD and lead to cellular aggregates with different morphologies. **b** Model of tau domain structural rearrangement and subsequent aggregation. Inert tau monomer (left) has a propensity to form a relatively collapsed conformation, which buries aggregation-prone elements. In the presence of disease-associated mutations, proline isomerization events, or certain splice isoforms, the equilibrium is shifted to disfavor local compact structure. This exposes the aggregation-prone elements and enhances aggregation propensity, leading to subsequent tau pathology. Structural models are shown in cartoon representation and are colored according to repeat domain as in Fig. 1. The aggregation-prone element is colored in blue. **c** Schematic of tau aggregation pathway for the formation of different strains. Soluble inert tau is shown as a cartoon highlighting local structures surrounding repeat domains, seed-competent monomer highlights structural rearrangements surrounding the aggregation-prone elements and fibrils are shown as an array of ordered monomers. Tau domains are colored as in Fig. 1

Sanders et al. also observed that isolation of strains from patients with identical neuropathological diagnoses, revealed considerable diversity within certain neuropathological diagnoses (Fig. 4a) [104]. That is, a given neuropathological diagnosis could be additionally sub-typed by strain analysis. This has raised the provocative question of whether the "ground truth" of neuropathological diagnosis is sufficient to properly define tau strains.

Expanding on the initial observations, Kaufman et al., isolated and characterized 18 tau strains that originated from either recombinant protein, tauopathy mice, or human brain [67]. The newly isolated strains were distinguished by multiple methods including inclusion morphology, detergent solubility, seeding, proteolytic digestion, and toxicity. After analyzing the distinct patterns of neuropathology produced upon inoculating PS19 mouse brains with the 18 strains, the authors concluded that strain diversity could account for all of the major neuropathological features associated with distinct tauopathies, including unique intraneuronal tau accumulation, distinct patterns of regional vulnerability, and rates of progression. In summary, evidence from inoculation studies in cell and mouse models, and analyses of strain content of human brain tissues strongly supported a model in which tauopathies can be understood as diseases caused by diverse strains [105, 125]. This raised the questions of how strains arise, and what assemblies account for them.

## Mechanisms of template formation and self-assembly

In recombinant form, or when expressed in non-diseased cells, tau is very stable and does not readily aggregate [38]. Early analyses of tau structure suggested that it does not adopt a stable folded conformation but rather is intrinsically disordered [12, 21, 114]. Given that tau encodes sequence elements that mediate self-assembly, a key question is how these elements are controlled so that aggregation only occurs under certain circumstances. Recent isolation and characterization of distinct pools of tau monomer, some with properties of seeding and self-assembly, and others without, indicate that tau adopts structure surrounding the elements that mediate aggregation (Fig. 4b) [16, 86]. A framework based on local structures that engage aggregation-prone sequences could explain tau’s stability and inability to aggregate in the absence of inducers.

Prior studies have indicated that the repeat domains contain local structure surrounding conserved KXGS and PGGG motifs [5, 64, 87]. The KXGS motifs are located within the middle of each repeat domain and are thought to be important for microtubule binding [11].The PGGG motifs which stabilize beta-hairpin conformations are located at the end of each repeat and are also immediately adjacent to aggregation-prone elements. Disease-associated mutations are enriched at these sites just upstream of these aggregation-prone elements (i.e. ^275^VQIINK^280^, ^306^VQIVYK^311^ and ^337^VEVKSE^342^). The Joachimiak lab has characterized the sequences surrounding the VQIVYK aggregation-prone element, finding that disease-associated mutations upstream from this element drive aggregation of tau by disrupting the protective structure (Fig. 4b) [16]. Thus, the formation of protective structures at these different amyloid-forming sites limits tau self-assembly and the transient nature allows it to be compatible with extended conformations necessary for microtubule binding [69]. Interestingly, recent work on tau under liquid–liquid phase separation conditions from the Zweckstetter lab proposed that stabilization of local structures around the KXGS motifs may promote oligomerization [5] but their capacity to drive aggregation remains unknown. Thus, local structure engaged by different sequence elements within tau may have opposing effects on the aggregation propensity. Indeed, recent work on alpha-synuclein has shown that engineering a specific beta-turn to adopt different geometries can have anti- and pro-aggregation properties, and thus the details of the beta-turn conformation are important for regulating aggregation [1]. Future work on tau will reveal details for how the specific conformations of beta-turns at these sites modify aggregation.

Initiation of tau aggregation in vitro requires the addition of preformed tau seeds or incubation with polyanions such as heparin [47, 94], octadecyl sulfate [17], RNA [47, 65], or arachidonic acid [70, 130] that disrupt these structures. Heparin appears to interact with defined sequences within the second tau repeat (RD2) [112, 136] and stabilizes an unfolded conformation of tau [30, 31, 112]. Thus, binding of polyanions to positively charged residues in the repeat domain may preferentially expose sequences that promote oligomer assembly during the lag phase followed by the elongation phase adhering to a classical nucleation mechanism [8, 109].

Tau monomer that is otherwise inert has the capacity to adopt stable aggregation-prone conformations that self-assemble and initiate aggregation upon induction in vitro and in the setting of disease states [110]. Structural analyses comparing inert vs. seed-competent monomer revealed preferential exposure of aggregation-prone sequence elements in seed-competent tau monomer, which then can act as a nucleus to promote elongation [16]. Furthermore, the seed-competent form of tau isolated from distinct tauopathies has been observed to encode distinct subsets of strains, which indicates a possible ensemble of aggregation-prone monomer conformations that have the capacity to adopt and propagate distinct fibrillar conformations (Fig. 4c) [110]. The idea that tau monomer alone can drive its own assembly, and, indeed can serve as a template to form structural polymorphs is not widely accepted, although additional recent work on tau [88], and Sup35, a yeast prion protein [92, 111], is consistent with this idea. Indeed, cryo-EM images of different filament conformations from individual patients reveals that the monomeric unit of tau in the fibrillar core of each polymorph is unique, however, subtypes have been observed in fibrils isolated from AD, CTE, CBD patients which might suggest some variation even within a disease [7, 32–34, 36, 135]. It remains unknown how tau might adopt these distinct monomeric conformations to yield oligomers and eventually fibrillar structures, but differential utilization of regulated local amyloid-forming sequences provides a testable model. A more detailed structural understanding of these initial conformational changes in tau monomer may be critical for identifying novel strategies for diagnosis and treatment.

## Cryo-EM structures of patient-derived fibrils

**Fig. 5:**
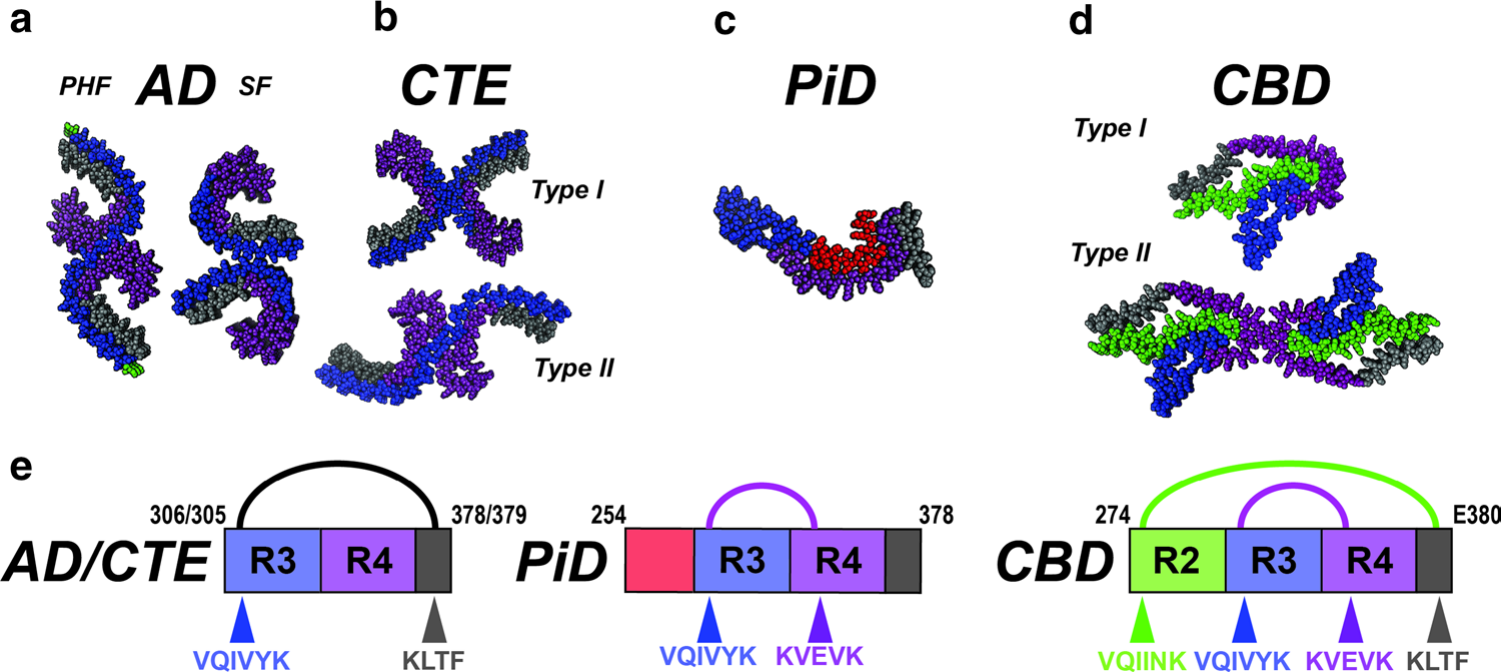
Unifying themes for diverse tauopathy fibrils. **a**–**d** Cryo-EM structures of tau fibrils isolated from AD-PHF, AD-SF, CTE (Type I and II), PiD and CBD (Type I and II). The structures are shown in spacefill representation, colored according to the repeat domains as in Fig. 1 and viewed down the fibril axis. **e** Schematic illustrating key contacts involving aggregation-prone elements observed in the different structures. Amino acids of each fibril are shown as a schematic and colored as in Fig. 1. Amino acids (including aggregation-prone elements) are colored according to the repeat domain and location indicated by an arrow. The linkage between contacts observed in AD/CTE, PiD and CBD are indicated by semi-circles and are colored black, magenta and green. The residues that comprise the amyloid structures are shown in the cartoon schematic

The Scheres, Goedert and Fitzpatrick laboratories have now used cryo-EM to describe in atomic detail the core structures of tau fibrils extracted from AD [33, 36], CBD [7, 135], Pick’s disease [32], CTE [34] and recombinant fibrils created by heparin induction [134]. Biochemical purification of insoluble filaments first allowed creation of electron micrographs [26], and methodological gains in cryo-EM now provide a glimpse of their core structure. Initial work involved classical paired helical filaments (PHFs) and straight filaments (SFs) from AD brains [36]. The PHF and SF are derived from a related C-shaped protofilament encompassing repeats 3, 4 and R’ (Fig. 5a; residues 306–378) as part of the core, but two different modes of assembly into fibrils (Fig. 5a). Subsequently, a related C-shaped conformation to AD-PHF/AD-SF that also encompassed repeats 3, 4 and R’ (Fig. 5a; residues 305–379) was observed in CTE fibrils, with two possible with packing arrangements between the two protofilaments defined as Type I and Type II (Fig. 5b) [34]. Interestingly, the authors observed an unexplained density, suggestive of a ligand within the core. Subsequently the structure of a PiD fibril revealed a flatter and more extended fibrillar shape that utilized repeats 1, 3, 4 and R’ (Fig. 5c; residues 254–378) [32]. Finally, two groups independently determined cryo-EM structures of CBD fibrils, revealing a conformation that utilizes repeats 2, 3, 4 and R’ (Fig. 5d; residues 274–380) [7, 135]. The CBD structures partitioned into two types: Type I with a single protofilament and Type II with two protofilaments related by a C2 symmetry (Fig. 5d). The monomer conformations were similar in each type, but the extent of ordering was different between the two types. As for CTE, the CBD fibril also had an unexplained density coordinated by basic residues. In addition to tauopathy-derived fibrils, cryo-EM structures for heparin-induced tau fibrils are described [134], which appear coated with heparin, suggesting that it plays a stabilizing role [36]. The stark differences between the disease- and heparin-derived fibrils raise obvious questions about the biological relevance of recombinant forms, especially for conformation-based therapies. Thus, the diverse tauopathy fibril conformations revealed by cryoEM supports our proposed model of tau strains and its unambiguous link to disease. Although cryo-EM structures have rightfully captured the field’s attention, this method has important limitations: a relatively small number of brain samples can be studied, and only after extensive purification of fibrils from large quantities of brain material. Further, there is no structural information for residues outside of the amyloid core that could contribute to strain formation, and only large detergent-insoluble filaments have been successfully imaged, which may not represent the critical tau oligomers.

## Molecular interactions in tau structural polymorphs

It is now possible to define unifying and/or distinguishing interactions that govern tau aggregation in fibrils. Aggregation-prone sequence elements mediate key interactions that appear to kinetically drive the formation of fibrillar folds in each of the tauopathy-derived fibrils. By contrast, recombinant heparin-induced tau fibrils lack these interactions. In the tauopathy fibrils, the remaining amino acids also segregate into defined clusters that create favorable interactions. These residues likely contribute less to the kinetics that determine the fold but may guide the final fibril conformation. Unlike globular proteins, fibril cores bury a mixture of polar and nonpolar interaction clusters leaving large, poorly interacting regions. This suggests multiple contributions to stability and specificity, and further a role for biological “tuning” to regulate functional amyloids. The importance of the nonpolar interaction clusters is highlighted by the central role of the VQIVYK aggregation sequence across all the published cryo-EM fibril conformations, including synthetic fibrils. Interactions of the VQIVYK sequence element vary, but common themes arise: three residues (V306, I308, and Y310) form essential contacts to either ^373^K**L**T**F**RE^378^ (AD, CTE, Fig. 5e) or ^337^**V**E**V**KSE^342^ (PiD, CBD, Fig. 5e), defined by discrete nonpolar-X-nonpolar amino acid patterning. In the case of the CBD fibril, the VQIINK amyloid motif interacts with ^373^K**L**T**F**RE^378^ (Fig. 5e) mimicking the VQIVYK interaction observed in the AD and CTE fibrils. Structures of isolated VQIVYK hexapeptides showed homotypic contacts between V306, I308 and Y310 and neighboring peptides. This highlights the propensity of these three residues to interact with themselves or form heterotypic contacts to other amyloidogenic motifs with defined spacing to drive fibrillization. While the AD-PHF, AD-SF, and CTE fibril structures are similar overall, and appear to be stabilized by similar contacts, the CBD and PiD structures differ, while still utilizing VQIVYK sequences identically (Fig. 5e). The comparison of tau fibril structures highlights the importance of nonpolar contacts for amyloid motifs, and how variation in their interactions stabilize similar (AD, CTE) and different (CBD, PiD) conformations. We hypothesize that nonpolar contacts may modulate aggregation and stabilize interactions that define the structural variants. Finally, considering the role of local structural elements in modulating aggregation, we propose that differential exposure of amyloid motifs may predetermine which contacts form thus play an important role in the kinetics of aggregation. This would constitute a series of structural "branches" defined by modular heterotypic interactions between amyloidogenic sequences that governs strain conformation, and ultimately disease manifestation.

## Therapeutic approaches

Tau is now considered the critical target in treating Alzheimer’s disease and other tauopathies [24, 59, 124]. The novel insights afforded by understanding tau strain diversity in tauopathies and in vitro models of aggregation must now be considered strongly in therapeutic development. For example, the first small molecules tested were methylene blue and its derivative LMTM which interfere with tau fibrillization in vitro and in animals reduced tau deposition [2, 3, 84, 131]. Unfortunately, in phase 3 trials testing LMTM in AD patients, there was no reduction in rate of decline of cognition compared to placebo [42]. We now recognize that in vitro and AD-derived fibrils have very different topologies and would likely be affected in different ways by a small molecule inhibitor of fibrilization. Thus, we must probably use models that mimic tau strains in tauopathy to properly evaluate small molecule aggregation inhibitors.

Because phosphorylation of tau fibrils was described in pathology, it was hypothesized that this drives tau pathology and thus that inhibition of tau kinases such as GSK3b would reduce disease. In preclinical mouse models, tideglusib, a small molecule targeting GSK3b reduced tau phosphorylation levels [108], but in subsequent phase 2 trials in AD patients, it failed to produce a clinical benefit [81]. Arguably, this failure may have been due to insufficient selectivity against other kinases [14] or non-optimal isoform selectivity [127]. However, it remains unclear whether phosphorylation is important in the initiation of aggregation.

Based on compelling cell and animal data that supports a model of trans-cellular propagation of pathology, passive and active vaccines to reduce tau pathogenicity are now in advanced human trials. Two active immune strategies are being tested. The first strategy, AADvac-1 is based on an epitope encoding residues 294–305 just preceding the VQIVYK amyloid motif[71]. The antigen is conjugated to a keyhole limpet hemocyanin carrier [91] is in Phase 2 trials clinical trials NCT02579252. Different tau strains feature this region either immediately outside the filament core or embedded within the amyloid segment. Thus, it will be informative to learn whether these differences in accessibility affect therapeutic outcomes. A second active vaccine, ACI-35.030, based on a multicopy synthetic phosphorylated peptide embedded in liposomes is now in Phase1b/2a. This strategy relies on the contribution of phosphorylated tau to seeding and trans-cellular propagation [120]. Numerous passive vaccines are in clinical trials [113]. A monoclonal antibody against the N-terminus of tau reduced pathological tau in a P301S mouse model [133]. A humanized version (ABBV-8E12/Tilavonemab) is in phase 2 for patients with early AD, after phase 2 for PSP was discontinued. Other anti-tau antibodies are in various stages of the clinical pipeline including Semorinemab, BIIB076, Gosuranemab, JNJ-63733657, Zagotenemab, and Bepranemab. Development of antibodies that are specific for tauopathy strains will likely be essential for optimal treatment of disease, and critically, vaccines will primarily target extracellular tau.

While vaccines to prevent trans-cellular propagation and small molecules that directly bind tau to inhibit aggregation will likely depend to some degree on tau strain identity, there are still many other approaches, especially those that target tau gene expression [124], which may work regardless of strain identity. While these are beyond the scope of this review, they offer important additional therapeutic opportunities.

## Strains in diagnosis of tauopathies

The recent development of tau binding agents has enabled the study of tau pathology onset and progression in patients with tauopathies. The description, over a decade ago, of [18F] FDDNP as a tracer with affinity for the tau lesions in AD catalyzed the expansion of markers in this category [121]. Broadly, there have been two iterations of tau ligands [93, 123]. The first-generation ligands include [18F] THK 5317, [18F] THK5351, [18F] Flortaucipir (previously known as AV1451/also-T-807), and [11C] PBB3. The second generation include [18F] MK 6240, [18F] RO-948, [18F] PI-2620, [18F] GTP1, [18F] PM-PBB3, [18F] JNJ-311 and its derivative [18F] JNJ-067 and were developed either based on existing structures of the first generation to improve specificity or represent new entities[123].

Virtually all published data from in vivo studies involves the first generation of tau PET ligands. These data have generated considerable excitement for the utility of this signal, but they have illustrated shortcomings with regard to off-target binding. Notably, several of the first-generation ligands bind monoamine oxidase isoforms [28, 77]. The substantial overlap in clinical presentation among primary tauopathies and other neurodegenerative syndromes further complicates the diagnostic utility of these agents. For example, Flortaucipir, THK 5317 and THK5351 are seen as useful for segregating cognitively normal individuals from patients in the CBS/PSP spectrum, yet have off-target binding in regions recognized to be harbingers of pathology, such as basal ganglia [78, 122]. Furthermore, several studies report intra-individual variability of tracer binding that is discordant with expected patterns of neuropathology [103, 132].

The lack of detailed study of the tau strain selectivity of each tracer has further complicated head-to-head comparisons [102]. The THK family, PBB3 and AV1451 reportedly bind NFTs, ghost tangles and neuritic plaques, and while AV1451 has relatively low affinity to SFs in CBD and PSP, the opposite is true for PBB3 and THK5351 [78]. If these differences in binding properties derive from conformational differences in tau, this would be very important. When viewed from the perspective of tau strains, it is not at all surprising that tau tracers with different binding sites on fibrils could conceivably detect alternative conformations of tau. The novel cryo-EM structures of filaments from different tauopathies may thus represent an important step toward the design of more specific tau tracers that allow the diagnosis of primary and secondary tauopathies by binding to strain-specific regions of tau fibrils.

## Outlook: strains in therapy and diagnosis

Awareness and knowledge of tau strains must guide both therapy and diagnosis. Specifically, unique conformations of tau will determine efficacy of small molecules in positron emission tomography (PET) and immunotherapy. For example, it is now well recognized that some tau PET ligands, while effective in certain tauopathies, are largely ineffective for others [78]. For immunotherapy, it seems likely that without determination of epitopes specific or common to various diseases, it will not be able to provide a universal treatment. Indeed, we should expect that therapies will have efficacy limited to subsets of tauopathy patients. We view this early stage of understanding as similar to the role that cancer genetics has played in personalizing therapy. The future seems very bright for similar successes in treating tauopathy, likewise we should not be discouraged by the fact that some patients will respond better than others. It will become ever more important to define the strains responsible for pathology antemortem, and preferably before symptoms arise in the first place.

## Key concepts

**Nucleation**: Event which generates a conformational state that is capable of propagation.

**Propagation**: Refers to the templated misfolding.

**Spread**: The movement of seeds between cells and brain regions.

**Amyloid**: Long, unbranched protein fibrils that display cross-beta fiber diffraction when examined by x-rays.

### **Seed**: 

(Noun) a conformational state that is capable of templating. Seeds may be generated by templated misfolding from pre-existing amyloid fibrils.

(Verb) to template misfolding.

**Infectious**: Likely to be transmitted to cause disease.

**Structural polymorph**: Distinct stable protein conformations (i.e.fibrils) adopted by a single protein sequence.

**Strain**: A unique prion conformation that replicates faithfully in living systems and confers specific biological effects.

**Prion**: A structured protein assembly that self-replicates in living systems and whose conformation controls its biological activity and potential for transmission between individuals.
